# Top-Down but Not Bottom-Up Visual Scanning is Affected in Hereditary Pure Cerebellar Ataxia

**DOI:** 10.1371/journal.pone.0116181

**Published:** 2014-12-29

**Authors:** Shunichi Matsuda, Hideyuki Matsumoto, Toshiaki Furubayashi, Hideki Fukuda, Masaki Emoto, Ritsuko Hanajima, Shoji Tsuji, Yoshikazu Ugawa, Yasuo Terao

**Affiliations:** 1 Department of Neurology, The University of Tokyo, Tokyo, Japan; 2 Department of Neurology, School of Medicine, Fukushima Medical University, Fukushima, Japan; 3 Segawa Neurological Clinic for Children, Tokyo, Japan; 4 Interfaculty Initiative in Information Studies, The University of Tokyo, Tokyo, Japan; Centre de Neuroscience Cognitive, France

## Abstract

The aim of this study was to clarify the nature of visual processing deficits caused by cerebellar disorders. We studied the performance of two types of visual search (top-down visual scanning and bottom-up visual scanning) in 18 patients with pure cerebellar types of spinocerebellar degeneration (SCA6: 11; SCA31: 7). The gaze fixation position was recorded with an eye-tracking device while the subjects performed two visual search tasks in which they looked for a target Landolt figure among distractors. In the serial search task, the target was similar to the distractors and the subject had to search for the target by processing each item with top-down visual scanning. In the pop-out search task, the target and distractor were clearly discernible and the visual salience of the target allowed the subjects to detect it by bottom-up visual scanning. The saliency maps clearly showed that the serial search task required top-down visual attention and the pop-out search task required bottom-up visual attention. In the serial search task, the search time to detect the target was significantly longer in SCA patients than in normal subjects, whereas the search time in the pop-out search task was comparable between the two groups. These findings suggested that SCA patients cannot efficiently scan a target using a top-down attentional process, whereas scanning with a bottom-up attentional process is not affected. In the serial search task, the amplitude of saccades was significantly smaller in SCA patients than in normal subjects. The variability of saccade amplitude (saccadic dysmetria), number of re-fixations, and unstable fixation (nystagmus) were larger in SCA patients than in normal subjects, accounting for a substantial proportion of scattered fixations around the items. Saccadic dysmetria, re-fixation, and nystagmus may play important roles in the impaired top-down visual scanning in SCA, hampering precise visual processing of individual items.

## Introduction

The cerebellum is intimately involved in coordinating skilled motor behavior, whereas impairment of the cerebellum is associated with cerebellar motor symptoms such as cerebellar ataxic movements, e.g. dysmetria. However, only recently has cognitive dysfunction come to be associated with cerebellar disorders. For example, disturbances of attention, executive functioning, and frontal lobe-like behavioral and affective alterations have been observed after focal primary cerebellar damage, resulting in a fairly common picture of cognitive and affective disturbances [1]. These cognitive impairments are generally thought to be caused by the cerebellar connections with the cerebral cortex [2]–[5]. The term ‘dysmetria of cognition’ has been proposed to refer to cognitive dysfunctions related to underlying cerebellar conditions [6]–[8].

However, it is uncertain whether dysmetria of cognition represents a condition caused by a similar mechanism to that of motor dysmetria or a distinct condition more related to cognitive processing and relatively independent of the condition of motor dysmetria. The simplest explanation would be that the cerebellum is involved not only in somatomotor function but also in oculomotor control. Cerebellar patients often present with a variety of oculomotor disorders including saccadic dysmetria [9]–[15], gaze-evoked nystagmus [9], [16], downbeat nystagmus [9], [17], and square-wave jerks [9], [18], which together can impair ocular fixation and compromise visual processing. Since most of the cognitive processing in daily life involves visual processing, the present study addressed how visual processing is impaired in cerebellar disorders.

Using an eye-tracking device, we recently showed that at least some part of the visual processing impairment in patients with spinocerebellar ataxia (SCA) may be accounted for by ‘dysmetria in oculomotor control,’ such as saccadic dysmetria and nystagmus, rather than dysmetria of cognition [19]. Our previous study, however, did not clarify the contribution of the two types of visual attentional mechanisms, ‘top-down instruction’ and ‘bottom-up salience,’ to deficits in visual cognition, [20]–[27]. People use these two types of attention when they search for the target among distractors in a search task [20], [28]. In the pop-out search task, where the target and distractors are clearly discernible, attention is captured by a visually conspicuous object (bottom-up salience) and subjects do not need to move their attention (or gaze) from one position to another. Consequently, the search time for detecting the target is independent of the number of items (the target and distractors) included in the display [21], [22]. On the other hand, in the serial search task, where the target has features very similar to the distractors, focused attention (or gaze) has to be allocated to each item serially from one to another (top-down instruction). As a result, search time increases with increasing number of distractors. Contrasting these two types of search in cerebellar patients should provide insights about impairments in these two types of visual attentional mechanisms, i.e. top-down instruction and bottom-up salience.

Saliency mapping is useful to study the relationship between search tasks and visual attentional mechanisms [27]. It is a conceptually simple computational model of focal visual attention that simulates bottom-up, image-based attentional deployment, accurately identifying the objectively outstanding areas in an image (bottom-up salience) [29], [30]. Analysis using saliency maps has so far been limited to studies of normal subjects [27], [29], [31]–[33] and has not previously been applied to patients with neurological disorders.

Previous studies of humans and animals with cerebellar damage have found impairment in visual search and suggested that the top-down attentional processes may be disturbed while bottom-up attentional processes are not [34]–[36]. However, these studies are controversial regarding the involvement of oculomotor movement [36], [37]. Moreover, these previous eye-tracking studies did not formally differentiate between serial and pop-out tasks. Thus, detailed analyses for inefficient top-down visual scanning are required to clarify the involvement of oculomotor control in the impairment of visual search.

In this study, we used two types of search tasks: serial search and pop-out search. To determine the visual attentional mechanisms in the two tasks, we employed a saliency map. Here, we hypothesized that cerebellar patients would need a longer time to search for a target than normal subjects, especially in the serial search task, which requires top-down visual processing. To evaluate the contribution of eye movements to the impairment of top-down visual processing, we also recorded eye movements during search tasks with an eye-tracking device, and compared the results with those of healthy subjects. To elucidate the causes of prolonged search time, we compared the parameters of visual scanning between SCA patients and normal subjects in the tasks that are disturbed in SCA patients: number of saccades, duration of fixation, amplitude of saccades, coefficient of variation (CV) of saccade amplitude, number of repeated fixations, and instability ratio of fixation.

## Materials and Methods

### Subjects

Eighteen non-demented spinocerebellar degeneration (SCD) outpatients who presented with pure cerebellar ataxia and 18 age-matched healthy volunteers (hereafter, normal subjects) participated in this study. Eleven SCD patients were genetically confirmed as spinocerebellar ataxia type 6 (SCA6) and 7 as spinocerebellar ataxia type 31 (SCA31) [38]–[41]. The Mini-Mental Status Examination (MMSE) [42] was used to exclude SCA patients with dementia from this study. The characteristics of the SCA patients and normal subjects are shown in Table 1. The mean ± SD age of the SCA patients was 64.1±12.0 years (range: 43–84 years) and that of the normal subjects was 64.6±11.1 years (range: 43–80 years), with no statistically significant difference between the two groups (unpaired *t*-test, p = 0.912). The median MMSE score of the SCA patients was 29 (range: 25–30) and that of the normal subjects was 29 (range: 26–30), again with no difference between the groups (Mann-Whitney U test, *p* = 0.845). The median duration of illness in the SCA patients was 9.5 years (range: 3–28 years). The median value of the International Cooperative Ataxia Rating Scale (ICARS) [43] was 58.5 (range: 5–73). Fifteen SCA patients showed gaze-evoked nystagmus and 11 showed downbeat nystagmus. None of them showed square-wave jerks.

**Table 1:** Characteristics of SCA patients and normal subjects.

	SCA patients	Normal subjects	*p*
N	18	18	
Male:Female	8:10	11:7	n.s
Age (years)			
Mean (SD)	64.1 (12.0)	64.6 (11.1)	n.s.
MMSE			
Median (range)	29 (25–30)	29 (26–30)	n.s.
Duration of illness (years)			
Median (range)	9.5 (3–28)		
ICARS			
Median (range)	58.5 (5–73)		
Fixation nystagmus (N)	15		
Down-beat nystagmus (N)	11		

MMSE: Mini-Mental Status Examination.

ICARS: International Cooperative Ataxia Rating Scale.

n.s.: not significant.

Written informed consent to participate in this study was obtained from all subjects. The procedure was approved by the Ethics Committee of The University of Tokyo and the study was conducted in accordance with the ethical standards of the Declaration of Helsinki.

### Eye-tracking analysis

The experimental setting was similar to that of our previous studies [19], [27], [44]. Subjects were seated with steady head position maintained by chin and forehead rests. The EyeLink 1000 system (SR Research, Mississauga, Ontario, Canada) was used to acquire ocular fixation position data at a sampling rate of 1000 Hz. This eye-tracking device can compensate for ocular fixation position shifts caused by head movements. Gaze data were acquired from the left eye. Tasks were created using SR Research Experiment Builder version 1.5.58 and images were presented on a Dell E173FPb monitor at 60 Hz. The distance between the screen and the subject was kept at 50cm, so that each image subtended a total visual angle of 38°×30°, with 0.85 cm on the screen corresponding to approximately 1° of visual angle. Prior to the experiments, the subjects performed a nine-point calibration procedure to map the ocular fixation position onto screen coordinates. The calibration was considered to be valid if the maximum spatial error was less than 1° and the average error was less than 0.5°.

### Behavioral paradigm: visual tasks

The following five different tasks were conducted: a simple reaction task, discrimination tasks (direction and color), and visual search tasks (serial and pop-out).

#### 1. Simple reaction task: somatomotor task

The subjects were instructed to press the button connected to the eye-tracking device as soon as a Landolt figure appeared at the center of the monitor. They were presented with a total of 10 images. The reaction time to press the button in this task was termed the simple reaction time, which was considered to reflect the speed of their somatomotor response.

#### 2. Discrimination tasks: cognitive tasks

In the discrimination tasks, every time the experimenter pressed the button, a Landolt figure appeared the center of the monitor.

**Figure 1:**
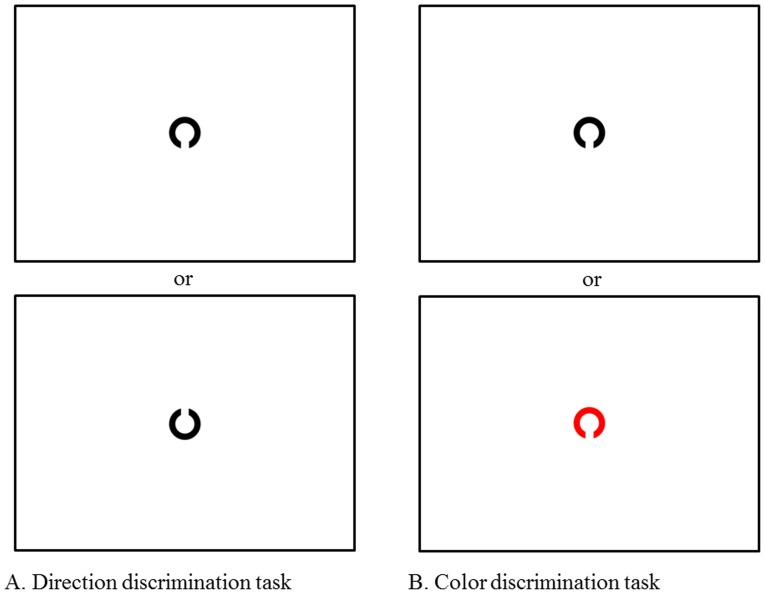
Discrimination tasks. A: direction discrimination task, B: color discrimination task. In the direction discrimination task, the subject was instructed to push the button connected to the eye-tracking device as quickly as possible only when an upward-facing Landolt figure appeared at the center of monitor. In the color discrimination task, the subject was instructed to push the button only when a red Landolt figure appeared. The direction and color discrimination times were measured by the following formula: discrimination time  =  total time – simple reaction time.

*2-1. Direction discrimination task:* The subjects were instructed to judge whether the Landolt figure was oriented upward (gap at the top) or downward (gap at the bottom; Fig. 1A). If the Landolt figure was oriented upward (target), the subjects were instructed to press the button as soon as possible, upon which the figure was extinguished. Ten figures in total were presented (5 upward and 5 downward). The direction discrimination time was calculated by the following formula: direction discrimination time  =  total time in this task – simple reaction time. The time to judge the direction of the Landolt figure in this task was considered to reflect the subject's cognitive speed of discriminating orientation.

*2-2. Color discrimination task:* In this task, the subjects were instructed to judge whether a Landolt figure appearing at the center of monitor was red or black (Fig. 1B). If the color of the Landolt figure was red (target), the subjects were instructed to press the button as soon as possible, at which point the image was extinguished. A session consisted of 10 image presentations (5 red and 5 black figures). Color discrimination time was calculated by the following formula: color discrimination time  =  total time in this task – simple reaction time. The time to judge the color of the Landolt figure was considered to reflect the subject's cognitive speed of discriminating color.

#### 3. Search tasks: cognitive and oculomotor tasks

In the visual search tasks, a display of a Landolt figures appeared when the subject pressed the button.

**Figure 2:**
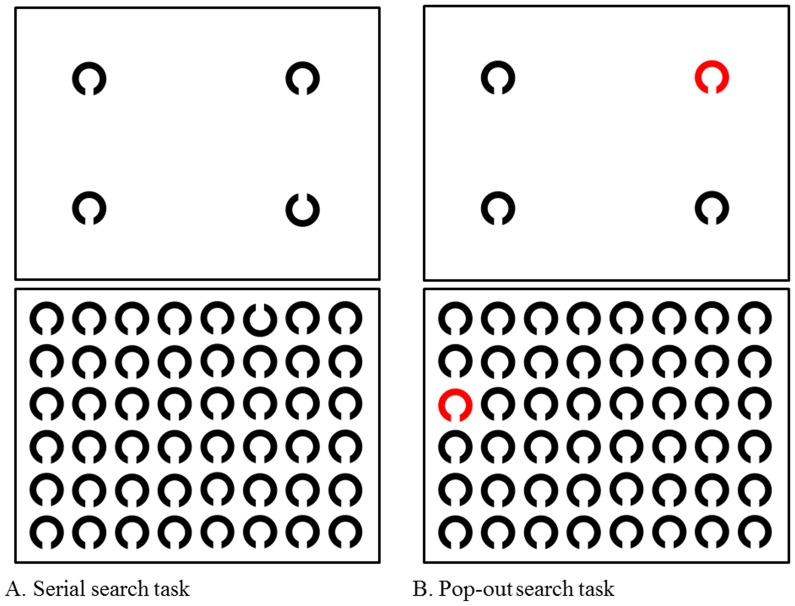
Search tasks. A: serial search task, B: pop-out search task. Upper panels show 4-item tasks and lower panels show 48-item tasks. In the serial search task, the subject was instructed to push the button connected to the eye-tracking device when he/she found an upward-facing Landolt figure. In the pop-out search task, the subject was instructed to push the button when he/she found a red Landolt figure. The serial and pop-out search times were measured by the following formula: search time  =  total time – simple reaction time.

*3-1. Serial search task:* The subjects were instructed to visually search for a single target Landolt figure out of distractor Landolt figures (Fig. 2A). The direction of the target Landolt figure was oriented upward whereas the other non-target Landolt figures were oriented downward. Each display contained a single target Landolt figure among multiple distracting Landolt figures. After the detection of the target Landolt figure, the subjects were instructed to gaze at it and to press the button, at which point the image was extinguished. They scanned a total of 10 similar images each for the 4- and 48-item tasks. Serial search time was calculated by the following formula: serial search time  =  total time in this task – simple reaction time.

*3-2. Pop-out search task:* The subjects were instructed to visually search for a single target Landolt figure out of distractor Landolt figures (Fig. 2B). The target Landolt figure was red while the non-target Landolt figures were black. After detecting the target Landolt figure, the subjects were instructed to gaze at it and to press the button, at which point the image was extinguished. As in the serial search task, the display contained either 4 or 48 items including the target (Fig. 2B). Subjects scanned a total of 10 similar images for each display size. Pop-out search time was calculated as follows: pop-out search time  =  total time in this task – simple reaction time. The search time to reach the target was considered to reflect the subject's oculomotor and cognitive function.

### Data analysis and statistical assessment

#### Saliency mapping

Using the method detailed in a previous report [27], we created saliency maps from the search tasks using MATLAB 2009a and custom software implemented in MATLAB [45], [46]. This software was designed on the basis of a bottom-up visual saliency model: the saliency mapping technique can predict human eye-fixation patterns successfully using the algorithms of Itti et al. [47]. The accuracy of saliency mapping predictions has been confirmed through comparison with data on human eye-fixation patterns while viewing the same scene [29]. For creating a saliency map, the visual input image is first decomposed into a set of topographic feature maps through several pre-attentive feature detection mechanisms (colors, orientations, contrast, and intensity). Different spatial locations then compete for saliency within each map, such that only locations which locally stand out from their surround can persist. All feature maps feed, in a purely bottom-up manner, into a master saliency map, which topographically codes for local conspicuity over the entire visual image [47].

#### Heat mapping

Heat maps, or graphical color-coded maps showing the distribution of ocular fixation positions, were created for each image using SR Research Data Viewer ver. 1.3.137. One heat map per image was created for each group, yielding a total of eight heat maps. To create a heat map, a two-dimensional Gaussian was applied to each of the fixation points. The Gaussian center was located at the ocular fixation position, while the width of the Gaussian was influenced by an adjustable sigma value (set at 0.8) in degrees of visual angle, and the height of the Gaussian was weighted by the duration of individual fixations. After the above process was applied to all fixation points, these Gaussians were normalized and overlaid in a color-coded fashion onto the original image.

#### Time in visual tasks and visual scanning parameters

The visual search times were measured in the serial and pop-out search tasks and were compared between SCA patients and normal subjects. The following data analyses were performed with programs in the SR Research Data Viewer ver. 1.3.137. To identify the causes of delay in search time in the visual search tasks (serial or pop-out search task), the number of saccades per second (n/sec), mean duration of fixation (ms), mean amplitude of saccades (degrees), CV of saccade amplitude, number of repeated fixations, and instability ratio of fixation were also measured as visual scanning parameters. The number of saccades per second was calculated as total number of saccades divided by search time. The duration of fixation indicates the mean duration of individual ocular fixations measured from the end of one saccade to the beginning of the next saccade. To count the number of repeated fixations, we selected the outline of the target Landolt figure (the circle surrounding the target Landolt figure) as the region of interest. Repeated fixations were defined as fixations which exited and re-entered this region. Based on the inspection of the eye movement records, the slow phase of nystagmus accounted for almost all of the instability of gaze due to impaired ocular fixation, and the ratio of gaze instability corresponds to the total time occupied by the slow phase of nystagmus within the total duration of the record. The instability ratio of fixation was therefore defined as the ratio of the duration of the slow phase in nystagmus to the total duration of the eye movement record. We followed the definition of the slow phase of nystagmus employed by previous studies, namely, slow oculomotor movements ranging from 5 to 10 degrees per second in an eye movement record [48], [49].

We performed the following statistical analyses: To compare the response times in the simple reaction and discrimination tasks (simple reaction time, direction discrimination time, and color discrimination time) between SCA patients and normal subjects, we used the unpaired *t*-test. Search times in the serial and pop-out search tasks were analyzed using the two-way repeated measures analysis of variance (ANOVA) with a within-subject factor: numbers of items (2 levels, 4 and 48 items); and a between-subject factor: subject group (2 levels, SCA patients-normal subjects). Post-hoc analyses were also conducted, if necessary, using the Bonferroni's method.

To investigate the difference in visual scanning parameters (number of saccades, duration of fixation, amplitude of saccade, CV of saccade amplitude, number of repeated fixations, and instability ratio of fixation) between SCA patients and normal subjects, we used a two-way repeated measures ANOVA with a within-subject factor: numbers of items (2 levels, 4 and 48 items); and a between-subject factor: subject group (2 levels, SCA patients-normal subjects). Post-hoc analyses were also conducted, if necessary, using the Bonferroni's method.

*P* values of less than 0.05 were considered significant (correction for multiple comparisons by Bonferroni's method: *p* <0.025). Statistical analysis was performed using the SPSS software package (ver. 16.0; SPSS Inc., Chicago, Illinois, USA).

## Results

### Saliency maps and heat maps

**Figure 3:**
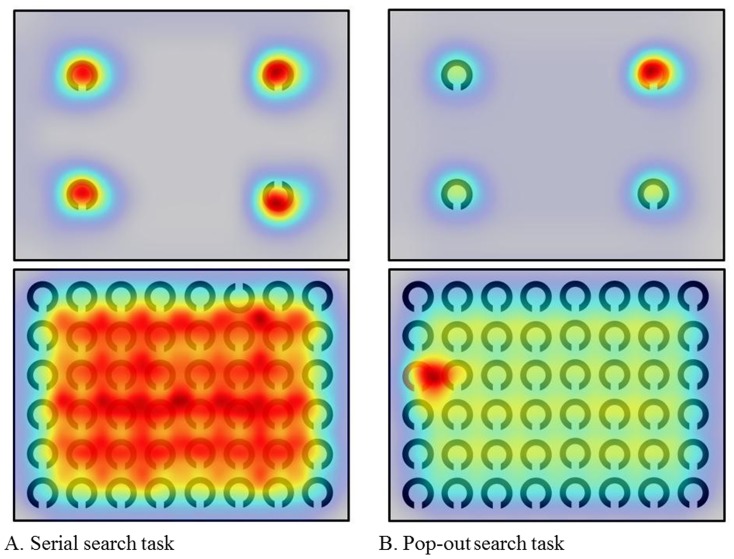
Saliency maps in search tasks. A: serial search task, B: pop-out search task. In the pop-out search task, the target red Landolt figure is clearly colored in red, suggesting that the subjects performed bottom-up visual scanning due to the high saliency of the target. In the serial search task, high saliency areas are uniformly distributed over the images and the target upward Landolt figure is completely masked, suggesting that the subjects performed top-down visual scanning due to the low saliency of the target.

**Figure 4:**
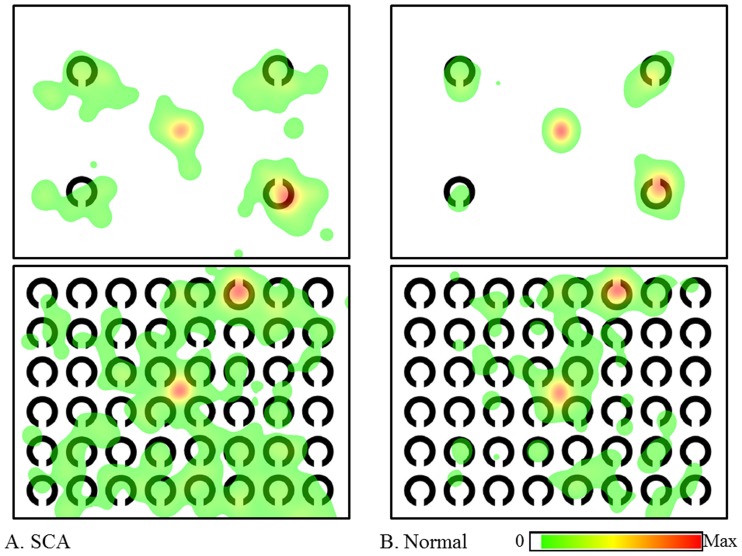
Heat maps in serial search tasks. A: SCA patients, B: normal subjects. In both 4- and 48-itemt tasks, the areas (colored areas) scanned by SCA patients were distributed more widely than those of normal subjects. The center was also colored because the starting eye-fixation position was located at the center of image.

**Figure 5:**
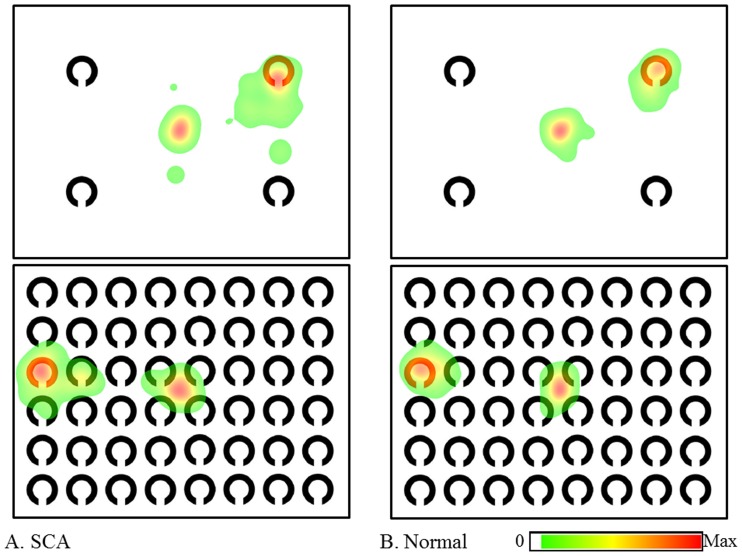
Heat maps in pop-out search tasks. A: SCA patients, B: normal subjects. In both 4- and 48-item pop-out search tasks, the areas (colored areas) scanned by both SCA patients and normal subjects were more localized than those in serial search tasks.

Fig. 3 shows the saliency maps in the search tasks. A saliency map color-coded according to the strength of salience was overlaid onto each image. Higher salience areas are depicted in red, intermediate areas in yellow, and lower areas in blue. Meanwhile, Figs. 4 and 5, respectively, show the heat maps color-coded overlaid onto each image according to the duration of gaze fixations in the serial search and the pop-out search tasks in 18 SCA patients and 18 normal subjects. Here, areas attracting longer fixations are shown in red, those attracting intermediate length fixations in yellow, and those attracting shorter eye fixations in green. The shortest durations (i.e., the lowest 5 percentile) were eliminated automatically as a default setting. Since the eye-fixation position was located at the center of the image at the start of each task, the center was colored despite the absence of items at this location. In all visual tasks, gaze fixations were distributed over a larger area in SCA patients than in normal subjects. In other words, the areas scanned by SCA patients tended to be enlarged relative to those by normal patients. Note that the gaze distribution almost coincided with the most salient regions of the image in the color discrimination tasks, meaning that the pop-out task actually drew attention (and gaze) to the most salient positions of the image, i.e. target positions (bottom-up visual scanning). In contrast, salient regions were more widely distributed throughout the images in the serial tasks, as were the positions of gaze fixation over the image. This reflects the fact that these images indeed require processing of items serially with gaze movements (top-down visual scanning).

### Impaired visual scanning in SCA: oculomotor trajectory

**Figure 6:**
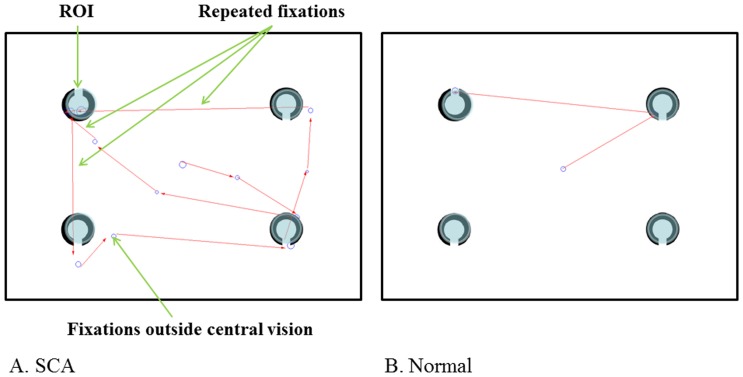
Representative eye movements. Eye movements in the 4-item serial search task are shown for an SCA patient (A) and a normal subject (B). In the SCA patient, the ocular fixation positions frequently did not fall exactly on the visual target, but landed at points somewhat removed from the target (saccadic dysmetria). In addition, we also observed something we term “repeated fixations (re-fixations)”. Here, the gaze initially captures the target item and then moves away before returning to the target item. To count the number of re-fixations, the target Landolt figure was selected as the region of interest.

We observed at least two abnormal eye movement patterns during the visual search tasks in SCA patients. One was saccadic dysmetria and the other was slow phase of nystagmus. Actually, the ocular fixation positions frequently did not exactly fall on the visual target, but landed at a point somewhat removed from the target, suggesting saccadic dysmetria (Fig. 6). A slow eye movement following a saccade was often encountered throughout the visual tasks, suggesting the slow phase of nystagmus. Careful visual inspection suggested that the enlarged scanned areas can be explained by oculomotor dysfunction such as saccadic dysmetria and gaze drift due to the slow phase of nystagmus. This suggests an impairment of visual scanning in SCA patients.

**Figure 7:**
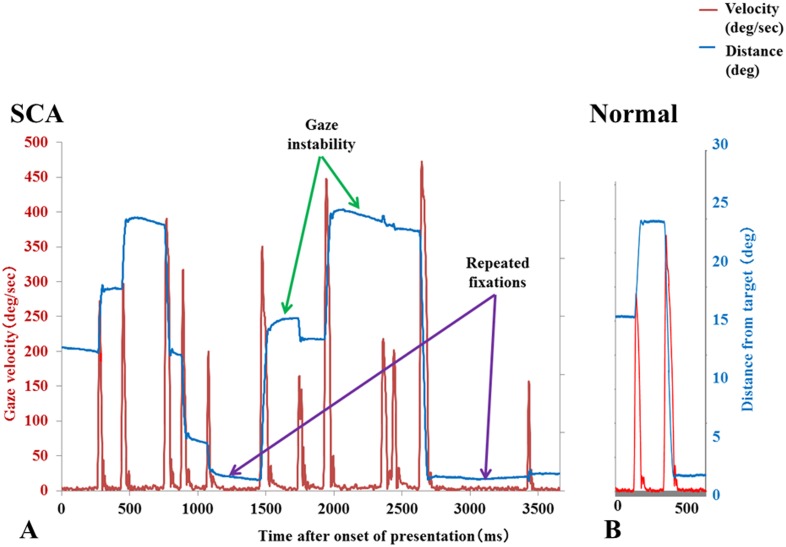
Oculomotor trajectories. Careful visual observation of oculomotor trajectories provides information on saccadic dysmetria, repeated fixations (re-fixations) and nystagmus. These figures show recordings of the gaze trajectory during visual search (abscissa: time; ordinate: the distance of gaze position from the target (zero deg. in the abscissa corresponds to the target position) [blue curve]; and instantaneous velocity of gaze [red curve]) in an SCA patient (A) and a normal subject (B). Note that with time the gaze eventually approaches the target (distance becomes zero at the right end of each figure) but for an SCA patient it takes more time, with the gaze alternately approaching and leaving the target. After approaching the target, the gaze leaves the target once and eventually comes back to the target later (repeated fixations). In addition, the gaze position shows slow drifts, representing the slow phase of nystagmus, even during presumed fixation, whereas this rarely occurs in normal subjects.

Analysis of gaze movements during the search task revealed that these abnormal gaze movements in SCA patients actually hampered visual processing of items. They frequently looked directly (within central vision) or relatively close to the items (target or distractors), but the distance of their gaze from the items again increased, with the gaze finally returning close to the target (Fig. 7A). In many instances, gaze fixation around the target was accompanied by slow drifting of gaze position, representing the slow phase of nystagmus. The same phenomenon occurred only rarely in normal subjects (Fig. 7B). As a result, SCA patients made significantly more saccades than normal subjects.

### Search time in visual tasks

**Figure 8:**
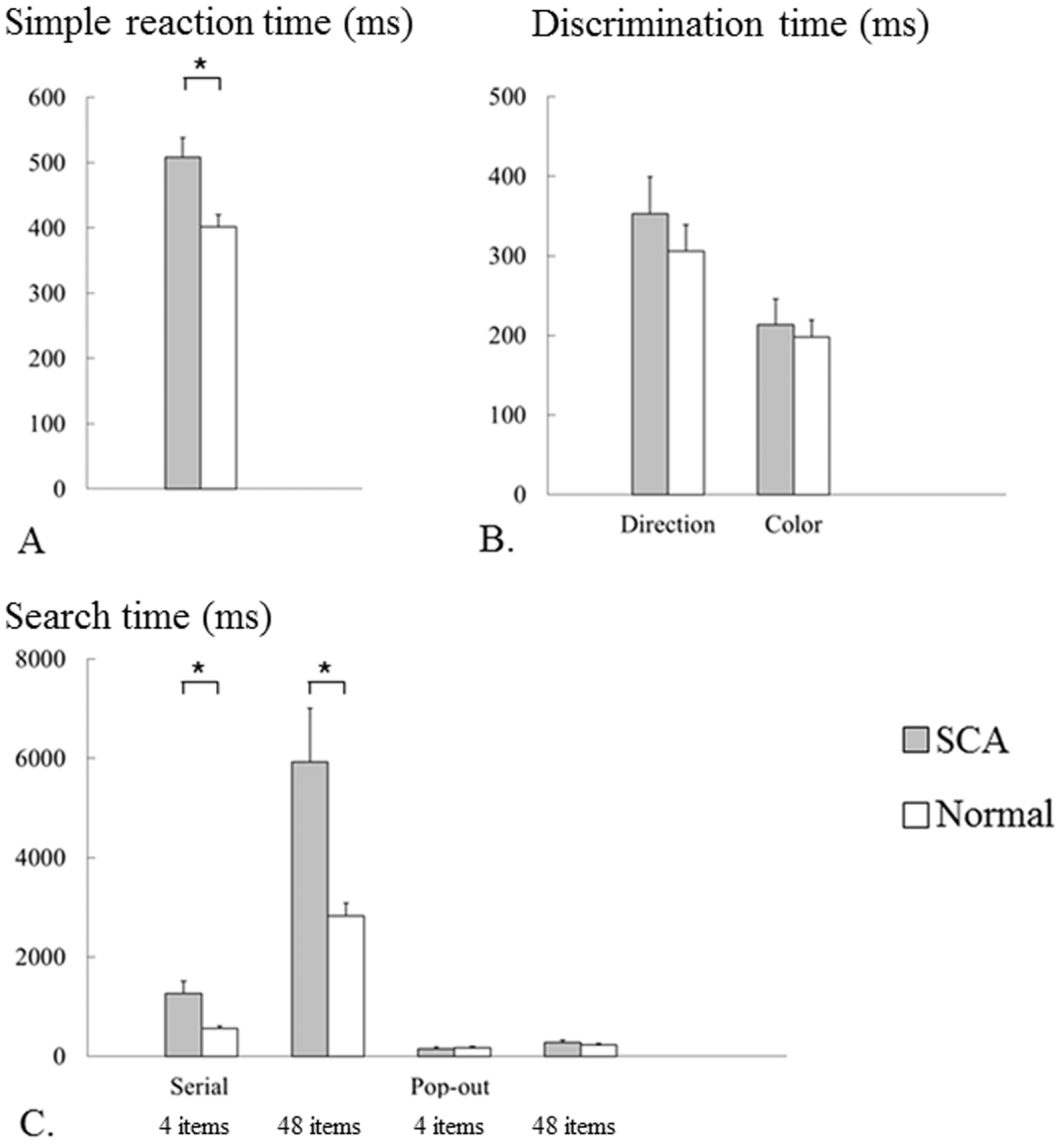
Search time of visual tasks. A: simple reaction time, B: direction and color discrimination time, C: serial and pop-out search time. The bars show the values of mean and standard error. Grey bars are for SCA patients and white bars for normal subjects. The simple reaction time was longer in SCA patients than in normal subjects. The direction and color discrimination time had no statistical differences. The 4- and 48-item serial search time was longer in SCA patients than in normal subjects, whereas the 4- and 48-item pop-out search time in SCA patients was almost identical to that in normal subjects. The serial search time was markedly longer than the pop-out search time in both SCA patients and normal subjects.

Table 2 and Fig. 8 show the performance in all five visual tasks. Simple reaction time was longer in SCA patients than in normal subjects (*p* = 0.004, Fig. 8A). The direction and color discrimination times were not different between SCA patients and normal subjects (Fig. 8B; direction: *p* = 0.411, color: *p* = 0.692). ANOVA and post-hoc analyses revealed that the serial search time in SCA patients was significantly longer than that in normal subjects in both 4-item and 48-item serial search tasks (Fig. 8C; test of within-subject effect: number of items × subject-group interaction, F_1_ = 5.293, *p* = 0.028; post hoc analyses: 4 items *p* = 0. 007, 48 items *p* = 0.009). Serial search time increased with the number of items for SCA patients and normal subjects (test of within-subject effect: number of items, F_1_ = 44.000, *p* <0.001). On the other hand, the pop-out search time in both the 4-item and 48-item tasks was not different between SCA patients and normal subjects (test of within-subject effect: number of items × subject-group interaction, F_1_ = 3.256, *p* = 0.080; tests of between-subjects effects: *F*_1_ = 0.051, *p* = 0.822). Pop-out search time also increased with the number of items for both groups, but with a much smaller increase than with the serial search task (test of within-subject effect: number of items, F_1_ = 28.370, *p* <0.001).

**Table 2:** Time in all visual tasks.

Time (ms)	SCA patients	Normal subjects	*p*
Simple reaction time	508.3±125.5	401.8±76.8	0.004*
Direction discrimination time	353.1±196.3	305.8±140.1	0.411
Color discrimination time	212.2±136.9	197.7±91.1	0.692
Serial search time			
4 items	1270.8±1019.3	564.2±180.1	0.007*
48 items	5926.4±4597.4	2821.9±1136.1	0.009*
Pop-out search time			
4 items	145.2±144.4	167.6±107.3	0.600
48 items	273.7±210.6	231.1±83.7	0.430

Data are shown as mean ± standard deviation; *significant difference.

### Visual scanning parameters

**Figure 9:**
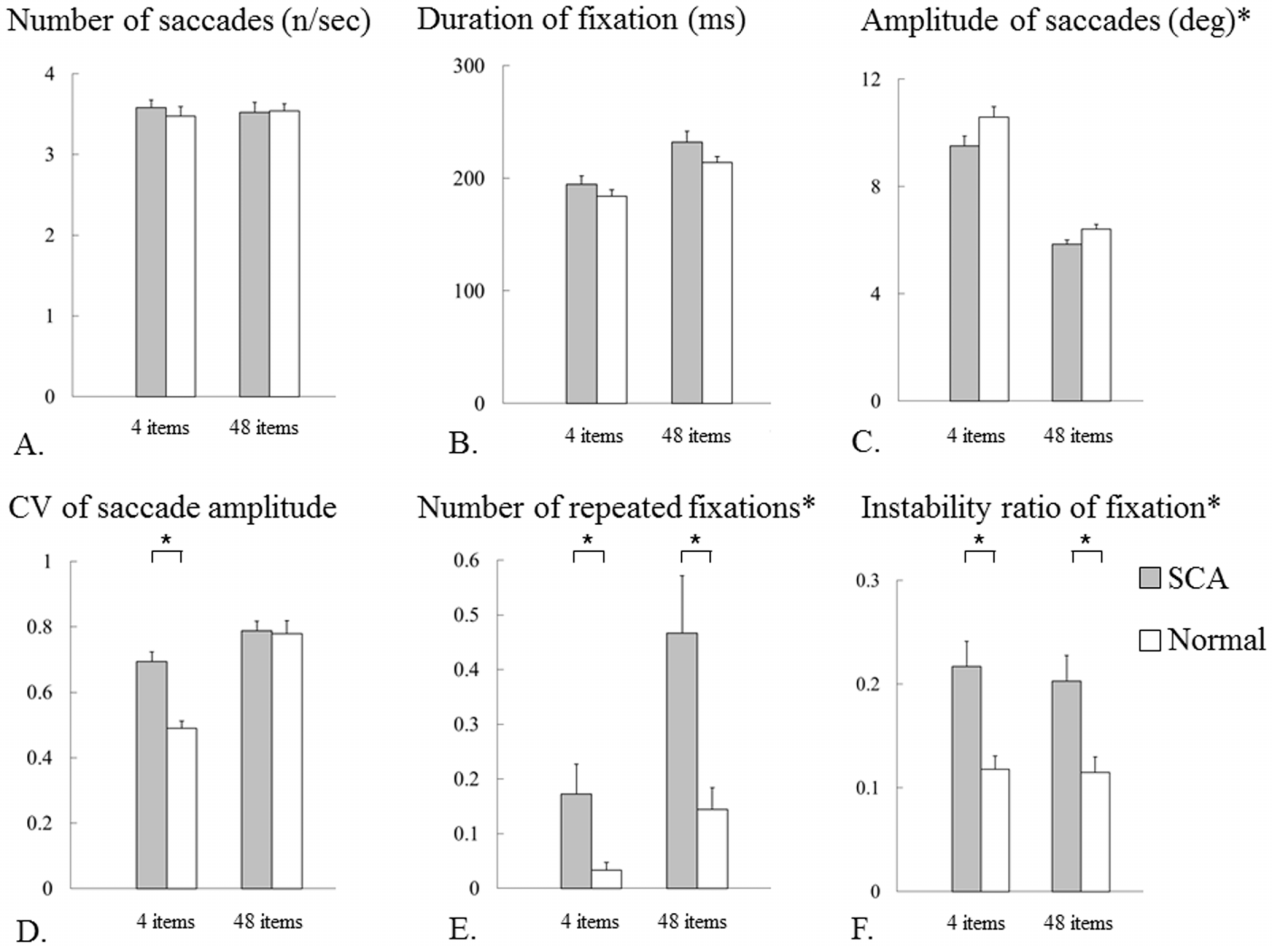
Visual scanning parameters in serial search tasks. A: number of saccades, B: duration of fixation, C: amplitude of saccades, D: coefficient of variation (CV) of saccade amplitude, E: number of repeated fixations, F: instability ratio of fixation. The bars show the values of mean and standard error. Grey bars are for SCA patients and white bars for normal subjects. The number of saccades per second and duration of fixation in SCA patients were identical to those in normal subjects for both 4- and 48-item tasks. Amplitude of saccades was smaller in SCA patients than in normal subjects for both 4- and 48-item tasks, although these differences were not statistically significant. CV of saccade amplitude was larger in SCA patients than in normal subjects only in the 4-item task. Number of repeated fixations and instability ratio of fixation were larger in SCA patients than in normal subjects for both 4- and 48-item tasks.

Saccade parameters during the serial search task were compared between the two subject groups to reveal possible causes for the impaired top-down visual scanning in SCA patients. Table 3 and Fig. 9 show the visual scanning parameters for SCA patients and normal subjects. The number of saccades per second in SCA patients was very similar to that in normal subjects (Fig. 9A; tests of within-subjects effects: number of items× subject group interaction, *F*_1_ = 1.278, *p* = 0.311; tests of between-subjects effects: *F*_1_ = 0.109, *p* = 0.744). The number of saccades did not change with the number of items for both groups (tests of within-subjects effects: number of items, *F*_1_ = 0.001, *p* = 0.979).

**Table 3:** Visual scanning parameters in serial search tasks.

	4 items			48 items		
	SCA patients	Normal subjects	*p*	SCA patients	Normal subjects	*p*
Number of saccades (n/sec)	3.6±0.4	3.5±0.5	0.479	3.5±0.5	3.5±0.4	0.924
Duration of fixation (ms)	194.4±32.2	184.0±24.4	0.281	231.8±40.8	214.1±22.0	0.114
Amplitude of saccades (degrees)	9.5±1.6	10.6±1.6	0.054	5.8±0.6	6.4±0.8	0.028
CV of saccade amplitude	0.69±0.12	0.49±0.09	<0.001*	0.79±0.13	0.78±0.16	0.873
Number of repeated fixations	0.17±0.23	0.03±0.06	0.019*	0.47±0.44	0.14±0.17	0.007*
Instability ratio of fixation	0.22±0.10	0.12±0.06	0.001*	0.20±0.10	0.12±0.02	0.004*

Data are shown as mean ± standard deviation; CV: coefficient of variation; *significant difference in post hoc analyses.

The duration of fixation in SCA patients was also almost comparable to that in normal subjects (Fig. 9B; tests of within-subjects effects: number of items × subject group interaction, *F*_1_ = 0.726, *p* = 0.400; tests of between-subjects effects: *F*_1_ = 2.289, *p* = 0.139). The mean duration of fixation increased with the number of items for both groups (tests of within-subjects effects: number of items, *F*_1_ = 62.206, *p* <0.001). However, these did not differ between the two groups as noted above and cannot explain the much longer search time in SCA patients. Thus, we looked for another reason why SCA patients make more saccades while searching for the target during visual search, with consequently longer overall search time.

First, saccadic dysmetria may have resulted from the impaired oculomotor control in SCA patients. The mean amplitude of saccades decreased with the number of items for both groups (test of within-subject effect: number of items, F_1_ = 200.825, *p* <0.001). The amplitude of saccades in SCA patients was significantly smaller than that in normal subjects (Fig. 9C; test of within-subject effect: number of items × subject-group interaction, F_1_ = 0.914, *p* = 0.346; tests of between-subjects effects: F_1_ = 6.705, *p* = 0.014). The CV of saccade amplitude in SCA patients increased with the number of items in both groups (test of within-subject effect: number of items, F_1_ = 35.809, *p* <0.001). It was significantly larger than that in normal subjects only in the 4-item serial search task (Fig. 9D; test of within-subject effect: number of items × subject-group interaction, F_1_ = 9.413, *p* = 0.004; post hoc analyses: 4items *p* <0.001, 48 items *p* = 0.875). These findings indicate that saccadic hypometria predominated in SCA patients, and that the saccade amplitude was more variable in SCA patients.

The number of re-fixations increased with the number of items for both groups (test of within-subject effect: number of items, F_1_ = 26.669, *p* <0.001). There were significantly more re-fixations in SCA patients than in normal subjects (Fig. 9E; test of within-subject effect: number of items × subject-group interaction, F_1_ = 5.450, *p* = 0.026). Similar re-fixations would also occur when the subjects were processing the distractors (see the analysis of oculomotor trajectory, Figs. 6 and 7). Of course, it must be noted that it is technically difficult to differentiate between ‘meaningful’ and ‘meaningless’ re-fixations.

As noted above, impaired gaze fixations, interrupted by slow drifts of gaze preceded by saccades, can also impair visual processing of items. The instability ratio of fixation, i.e. the proportion of time occupied by such impaired fixation, was significantly larger in SCA patients than in normal subjects (Fig. 9F), implying unstable ocular fixation in SCA patients (tests of within-subjects effects: number of items × subject-group interaction, *F*_1_ = 0.912, *p* = 0.346; tests of between-subjects effects: *F*_1_ = 11. 524, *p* = 0.002). The ratio did not change with the number of items in either group (test of within-subject effect: number of items, *F*_1_ = 2.218, *p* = 0.146).

To assess how saccadic dysmetria affected the spatial separation of the gaze from the target, the landing positions of the gaze was counted by setting the region of interest (a target Landolt figure) (Fig. 6). Postulating a range of central vision of 2° of radius ( = R) around the direction of the gaze, we counted the number of fixations which landed near each target within a certain distance during the visual search. In the 48-item task, the number of fixations made within a distance of R around the target (captured with central vision) was larger in SCA patients than in normal subjects (3.2±1.1 vs. 2.6±0.8, respectively; p = 0.070). Within a distance of 1.5R, the number was 5.7±2.6 for SCA patients and 3.8±1.2 for normal subjects (p = 0.010). In the 4-item task, the number of fixations made within a distance of R was 1.8±0.7 in SCA patients and 2.0±0.6 in normal subjects (p = 0.368), and within a distance of 2R, the number was 3.3±1.3 in SCA patients and 2.8±0.6 in normal subjects (p = 0.155), with no significant difference between groups. Within the distance of 3R, however, the number was 4.2±1.7 in SCA patients and 3.0±0.6 in normal subjects (p = 0.011). Thus, for both the 4- and 48-item tasks, the number of fixations around the target became larger for SCA patients as the distance from the target grew larger.

## Discussion

Our study suggests that the impairment of top-down visual processing in SCA patients mainly results from: 1) saccadic dysmetria resulting in larger separation of the landing locations of the gaze from the target, 2) re-fixation of the target (and possibly of distractors), and 3) impaired fixation due to slow drifts of gaze associated with nystagmus.

### Top-down visual scanning is markedly impaired in SCA

In this study, we compared the ability of SCA patients and normal subjects to perform top-down and bottom-up visual searches. Importantly, SCA patients needed a markedly longer search time than normal subjects in the serial search task. In contrast, SCA patients and normal subjects showed almost the same search times in the pop-out search task. Meanwhile, the response times in both the direction and color discrimination tasks were comparable between SCA patients and normal subjects. This reflects the fact that simple visual processing remains intact in SCA patients, due to the absence of eye movements. These findings suggest that SCA patients have problems searching for a target serially using a top-down attentional process, whereas scanning with bottom-up attentional process is not affected.

These results are consistent with a previous study in which search behavior was slower and less efficient in patients with cerebellar infarcts, especially for tasks requiring serial processing [37]. However, this is the first study to show the impairment of top-down visual scanning in patients with cerebellar dysfunction using saliency maps. We also analyzed what caused this impairment of search in SCA patients by monitoring eye tracking during the performance of visual search tasks.

### SCA patients must perform more saccades to detect a target with low saliency

Serial search time was longer in SCA patients than in normal subjects, suggesting that SCA patients have to make more saccades to detect a target due to the impairment of top-down visual processing. We explored the reason for the longer search time in SCA patients. The lack of difference in mean fixation duration between SCA patients and normal subjects excluded the possibility that SCA patients were not fixating long enough to extract sufficient information during each fixation. If anything, the mean duration of fixation tended to be longer in the SCA patients. Furthermore, in both the direction and color discrimination tasks, which test simple cognitive visual processing without eye movement, the discrimination times were comparable between SCA patients and normal subjects, suggesting that SCA patients retain the ability to judge the characteristic features of a Landolt figure. Therefore, the possibility of impaired simple visual processing in the absence of eye movements is less likely. The search time difference between SCA patients and normal subjects was much more prominent in the serial than in the pop-out task. These findings suggest that having to make more saccades during the course of visual search is strongly associated with the longer search time in SCA patients. This poses the question: Why do SCA patients make more saccades than normal subjects?

### Mechanisms of impaired visual processing suggested by gaze trajectories

Analyses of gaze trajectories during the serial visual search task suggests that making saccades itself prevented SCA patients from recognizing the visual items (target and distractors) efficiently, and they had to make more saccades to “see them better”. The most impressive result of the eye-tracking studies was that SCA patients frequently looked directly (within central vision) or relatively close to the items (target or distractors) outside central vision but within 5 degree of items, but still went past and away from them, and later returned to the items. What this suggests is that SCA patients could not somehow process the visual information of the items properly when they had to make eye movements, so that they needed a lot of repeated fixations to better perceive the items. These re-fixations are of special note because they indicate that SCA patients may be unable to properly process and interpret what they are seeing “on the first try”.

Analyses of gaze trajectories showed that saccadic dysmetria resulted in scattering of gaze fixations around the target, with larger spatial separation from the latter, hampering accurate visual perception of the target from distractors. In addition, during gaze fixation, the visual processing of items was substantially impaired by saccade drifts due to the slow phase of nystagmus or saccade dysmetria. This was often followed by corrective saccades or saccades representing the quick phase of nystagmus. Even when the gaze approached the target with central or parafoveal vision, saccadic dysmetria and impaired fixation disrupted the processing of items.

### Impairment of serial search relative to pop-out search and the contribution of eye movements

Recently, it has been proposed that the cerebellum contributes to cognitive functions, including attention [1], [6], [50]. While spatial shifts of visual attention are strongly associated with saccades (overt attention), attentional shift can also be made without eye movements (covert attention). Previous papers, using tasks that separately addressed overt and covert attention, consistently report that disturbed overt but normal covert shifts of attention in cerebellar lesions [34], [35], [50]. Therefore, the findings of our study agree with these papers, in that the serial search task requiring sequential (top-down) visual processing of items and gaze movement was affected in SCA patients, whereas the pop-out (bottom-up) search task was not.

Importantly, we also found that gaze movements during visual search provide a relatively good explanation for how the visual impairment occurred. By contrasting the serial and pop-out tasks, we clearly showed how the spatial shifts of attention that occur along with the gaze movements may impair the detection a target. We also showed how saccadic dysmetria (predominantly saccadic hypometria), re-fixations and nystagmus play important roles in the abnormal top-down visual scanning in SCA patients, resulting in imprecise eye movements, which hamper precise visual processing of central vision.

It must be acknowledged that the analysis of gaze trajectory does not provide a definite explanation of how impaired oculomotor control relates to the impairment of top down visual processing as demonstrated by prolonged search time. Top down visual processing may consist of three components: 1) a strategic component that determines how the subjects deploy their attention over a presented display to process the items visually, 2) an oculomotor component that determines how the subjects plan or program their oculomotor movements based on #1, and 3) a cognitive component that processed the visual information received during fixation. These parts are probably not independent of one another. That is to say, how accurately the gaze captures the presented items and how precisely the visual information taken in during fixation would in turn affect the strategic part, i.e., how the subjects plan subsequent eye movements. The presence of re-fixations suggests that even when the eye movements are made accurately enough to capture the items with central vision, the gaze can return to it, which suggests a deficit in the strategic component or the cognitive component of top-down visual processing. On the other hand, saccadic dysmetria and impaired fixation are believed to represent a deficit in the oculomotor component, which also affects cognitive processing by hampering the incoming visual information [19].

We must be careful, however, that it is not possible to conclude, based on the present results, whether the impairments of top-down visual scanning are only due to deficits in eye movements control or are the consequence of a specific problem in top down mechanisms. To solve this problem, we would need visual search tasks that requires top down mechanisms to be performed, only with attentional shifts but without eye movements [51].

## Conclusions

Using saliency maps, we showed that top-down visual scanning is impaired but bottom-up visual scanning remains intact in hereditary pure cerebellar ataxia patients. Saccadic dysmetria, re-fixations, and nystagmus may play important roles in the impairment of top-down visual scanning in these disorders, hampering precise visual processing.
